# Deep learning neural networks-based traffic predictors for V2X communication networks

**DOI:** 10.3389/frai.2025.1701951

**Published:** 2025-12-15

**Authors:** Marina Magdy Saady, Hatim Ghazi Zaini, Mohamed Hassan Essai Ali, Sahar A. El_Rahman, Osama A. Omer, Ali R. Abdellah, Shaima Elnazer

**Affiliations:** 1 Department of Electrical Engineering, Faculty of Engineering, Aswan University, Aswan, Egypt; 2 Computer Engineering Department, College of Computer and Information Technology, Taif University, Taif, Saudi Arabia; 3 Department of Electrical Engineering, Faculty of Engineering, Al-Azhar University, Qena, Egypt; 4 Department of Computer Systems Program-Electrical Engineering, Faculty of Engineering-Shoubra, Benha University, Cairo, Egypt; 5 Nile Higher Institute of Engineering and Technology, Mansoura, Egypt

**Keywords:** CNN, deep learning, optimizers, RNN, traffic prediction, V2X

## Abstract

Vehicle-to-everything (V2X) communication is a promising technology for enhancing road safety, traffic efficiency, and the availability of infotainment services in 5G networks and beyond networks. However, the effective sharing of traffic information remains a significant challenge. To address this, AI-based systems offer potential solutions. By predicting traffic patterns on dense networks, these systems can improve traffic management, mitigate congestion, increase network safety and reliability, and improve energy efficiency. This research investigates the application of Recurrent Neural Networks (RNNs) and Convolutional Neural Networks (CNNs) for accurate and efficient V2X traffic prediction. We explored the impact of various hyperparameters, including loss functions and optimizers, on the performance of these models. Our findings indicate that Gated Recurrent Unit (GRU) models, particularly with the Mean Squared Error (MSE) loss function and Adam optimizer, consistently outperform Long Short-Term Memory (LSTM) and Bidirectional Long Short-Term Memory (BiLSTM) models in terms of both accuracy and computational efficiency. For CNN models, the Rectified Linear Unit (ReLU) activation function, coupled with the Adam optimizer, demonstrated superior performance in terms of Root Mean Square Error (RMSE) and computational complexity. By comparing our results with existing literature, we highlight the advantages of our proposed models in terms of accuracy, efficiency, and robustness.

## 1 Introduction

Fifth-generation (5G) cellular systems and beyond are predicted to enhance quality of service (QoS), high throughput, improved network safety, increased capacity, low latency, and low cost. As the number of devices rises, so does the flow of information, making the network more difficult to manage and operate (Abdellah et al., 2022a; Sarker, 2022). For the 5G network, efficient and innovative methods are required to modify network protocols and manage resources for various services under multiple scenarios. Artificial intelligence (AI) is a leading technology in advanced intelligent technologies that allow intelligent and fast business process decisions, enhancing profitability and efficiency (Ahmed et al., 2023; Jiang et al., 2022).

Recently, AI technology has been utilized in 5G wireless networks to optimize the physical layer architecture, network management, complex decision-making, and resource allocation. Big data techniques offer a great chance to grasp wireless network essentials and better comprehend 5G cellular network performance (Abubakar et al., 2020; Hassan et al., 2023). Machine learning (ML) provides or predicts new entries in most AI applications. ML solves problems like wireless network optimization and attack detection (Brik et al., 2022).

Deep learning (DL) methods have proven robust for predicting network traffic and forecasting accuracy. DL algorithms based on neural networks (NNs) are promising solutions to improve prediction accuracy in data traffic flow. Many different types of NNs have been developed for various objectives; recurrent neural networks (RNNs) are made to process historical information or observations collected over specific periods; traffic patterns are an example of such observations (Sepasgozar and Pierre, 2022). A critical issue for traffic prediction is the accuracy of the forecasts to overcome the challenges of 5G mobile networks without further reducing the efficiency of the system's quality of service (QoS) (Abdellah et al., 2022a). Numerous techniques have been created to increase traffic, enhancing forecasting accuracy (Tsourdinis et al., 2022). Motivation for this study:

- Traditional RNNs have problems connecting temporally distant events.
- DL is a powerful method that can build accurate predictive models from vast amounts of unlabeled, unstructured data by instantly creating complicated statistical models based on their iterative output.
- DL-based BILSLTM and Gated Recurrent Unit (GRU) models better predict time series because they remember historical data. They optimize the learning technique during training iterations and outperform existing time series prediction approaches. For real-time traffic forecasts, adding more data makes the model smarter and better at estimating traffic volumes.
- Timely accurate traffic predictions improve network QoS.
- Monitoring and controlling network traffic.
- The anomalies in smart network traffic are high, leading to prediction problems.
- Improve network control and QoS requirements, manage resources, and detect security issues.
- Monitor network connectivity and activity for security and operational problems.
- Inaccurate ML analysis prevents accurate prediction.
- QoS optimization's computational difficulties.

Due to these limitations, this work aims to predict Vehicle-to-everything (V2X) network traffic. Healthcare, security, transportation, and medical emergencies require applications that efficiently use traffic resources. Predicting network traffic and bandwidth helps identify security and performance problems. Identifying the next steps in intensive remote patient monitoring requires a critical case to be reported to a healthcare organization within a certain timeframe. This situation results in varied information depending on the quantity and type of observations. DL techniques such as the Bidirectional Long Short-Term Memory (BiLSTM) and GRU predictors can forecast traffic volumes.

The main contributions to the proposed work are as follows:

- Long Short-Term Memory (LSTM), Bidirectional Long Short-Term Memory (BiLSTM), and Gated Recurrent Unit (GRU) have been tested for the designed models.
- A comparison was conducted between the proposed RNN models and traditional LSTM models (Abdellah et al., 2022a) regarding prediction accuracy.
- A one-dimensional convolutional neural network (CNN, temporal convolutional network) with three activation functions is tested.
- A comprehensive study for the proposed loss function model, including mean squared error (MSE), mean absolute error (MAE), and sum of squared errors (SSE).
- A comprehensive study was conducted for the proposed loss function model, including the Adam, Stochastic Gradient Descent with Momentum (SGDM), and Root Mean Squared Propagation (RMSprop) optimization techniques with a batch size of 16 and a learning rate of 0.1.

We evaluated prediction accuracy using root mean squared error (RMSE) and efficiency in terms of total Floating-point Operations Per Second (FLOPS) [MegaFLOPS (MFLOPS)].

## 2 Related work

Several studies have explored the use of RNNs for traffic prediction, including LSTM and GRU networks (Mahajan et al., 2024) applied LSTM networks for real-time traffic flow forecasting, achieving root mean square error (RMSE) values of approximately 1.23 for urban traffic data. In contrast, Kim et al. (2021) used GRU for highway traffic prediction and reported an RMSE of 0.85 under similar conditions. In Fitters et al. (2021), an architecture based on an LSTM network was investigated for predicting and focusing on irregular traffic flows. Abdellah et al. (2022b) used an LSTM network-based DL approach to forecast drone-based MEC energy consumption time series. Four cases examined accuracy as a function of learning rate. RMSE and MAPE were used to determine the best and highest average prediction accuracy (Abdellah et al., 2022b). Wang et al. (2022a) suggested a spatiotemporal study of mobile network traffic and reviewed current research. Time series similarity-based graph attention networks were also proposed. Using an LSTM network with modified hyperparameters, a DL model predicts short-term traffic speeds on a parallel, multilane arterial road in an emerging country such as Vietnam (Tran et al., 2022). An LSTM-based practical method for accurately predicting environmental movement to improve security decision-making and path planning was first presented in Wang et al. (2022b). Then, a risk assessment was used to plan local paths. Based on these correct predictions, the risk assessment is field-based.

In the domain of CNNs for traffic prediction (Cao et al., 2021), used a hybrid CNN-LSTM model for traffic speed prediction, achieving an RMSE of approximately 0.91 for highway traffic, which is considered competitive for deep learning models in traffic prediction. Similarly, in Ma et al. (2017) applied a simple CNN model on urban traffic datasets and reported an RMSE around 0.98 with Rectified Linear Unit (ReLU) activation.

In contrast, these studies have limitations in terms of V2X communication (Srivastava et al., 2014):

- LSTM models struggle to handle massive traffic flow data simultaneously with computing and distributed storage requirements.
- The current traffic forecast methods have been unsuccessful in addressing complex road segment association.
- The current traffic prediction algorithms are sensitive to illumination conditions.
- Recent studies have failed to model or develop dynamic traffic patterns for unstable environments.
- Most prediction methods address accuracy rather than timeliness.
- Existing machine learning (ML**)** methods disregard traffic network system complexity and heterogeneity.
- The current research with experiments not based on deep learning (DL) finally faced difficulties training a deep network due to complexity and time limitations.

To address these limitations in existing models, this study focuses on an essential issue for traffic prediction: the accuracy of forecasts to address 5G mobile network issues without reducing QoS system efficiency. Therefore, we address these design issues in this work by training alternative Deep Learning Neural Network (DLNN) architectures based on V2X packets-per-second data. These predictors don't require prior knowledge about the surrounding environmental conditions (channel statistics) and benefit from the excellent learning and generalization capabilities of DNNs. The proposed DL model will be built using LSTM, BiLSTM, and GRU, which are variations of the RNN, to solve the vanishing gradient problem. For CNNs, we will test the most powerful activation functions (ReLU, Tanh, and Sigmoid) reported in similar work. The prediction accuracy regarding root mean squared Error (RMSE) and Floating-Point Operations Per Second (FLOPS) will be assessed. The best predictors will be improving QoS demands, monitoring resource management, enhancing security, and other operational issues.

## 3 DL approach in V2X communications

Driverless and autonomous vehicles are becoming more popular because they are better for businesses and emergency services. These vehicles need constant sensor data for complex, high-speed operations and improved trajectory planning for these services. The car can use onboard sensor information for short-term trajectory decisions, but needs data from nearby vehicles for long-term decisions. Therefore, sensor data sharing is essential and requires reliable vehicle connectivity, subject to strict QoS requirements (Gao, 2022). Thus, modern wireless networks connect cars, people, infrastructure, roads, etc., via advanced communication technologies. Advanced communication technologies enable vehicle-to-everything (V2X) communication.

**Figure 1:**
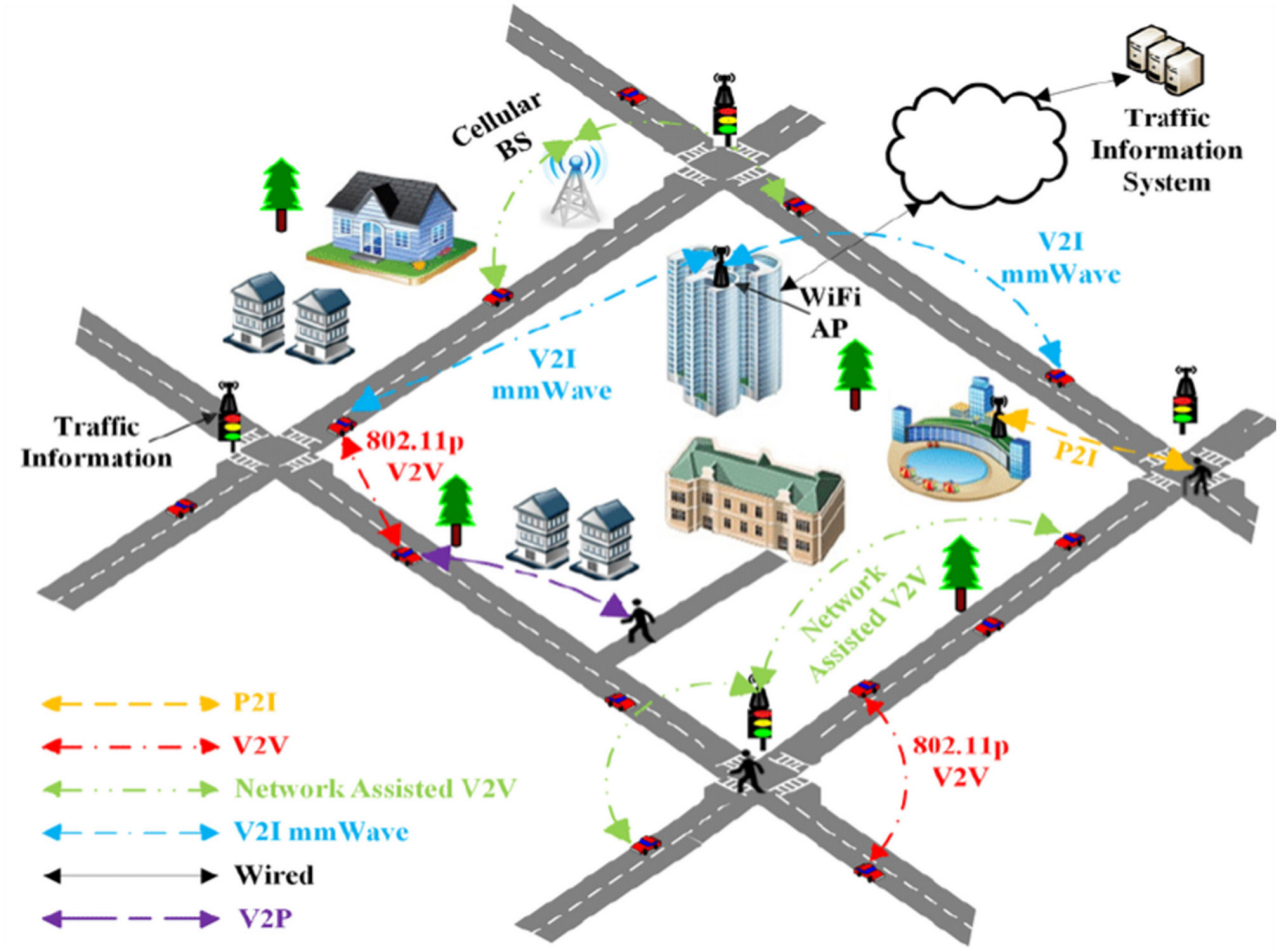
Vehicle-to-everything communications (Ullah et al., 2019).

V2X communication protocols and technologies allow vehicles to interact with roadways and users. V2X allows Vehicle-to-Vehicle (V2V), Vehicle-to-Infrastructure (V2I), Vehicle-to-Pedestrian (V2P), and Vehicle-to-Cloud (V2C) interactions. Figure 1 depicts the 5G V2X communication infrastructure. v2x communication and traffic growth cause long delays in mobility, economic growth, fuel costs, air pollution, and public health. Most intelligent traffic control methods are still in their infancy. Thus, traffic control must be thoroughly examined to test a new V2X-based smart traffic management theory on congested roads (Gao, 2022).

To overcome the issues with 5G mobile networks and prevent further degradation of the quality of service (QoS) system, numerous solutions are required to improve the precision of traffic feature prediction. DL algorithms have proved highly effective in predicting road traffic compared to statistical techniques (Storck and Duarte-Figueiredo, 2020). Deep learning techniques, including convolutional neural networks (CNNs), Recurrent Neural Networks (RNNs), and Hybrid Models, are widely utilized to predict various aspects of traffic, such as flow, congestion, travel time, and accident-prone zones. These models are capable of learning complex, non-linear relationships in data, making them ideal for traffic prediction, which involves a mix of dynamic factors, environmental conditions, and temporal sequences. Table 1 shows a comparison between RNNs and CNNs for traffic prediction in V2X communication.

**Table 1:** A comparison between RNNs and CNNs.

**Network**	**CNNs**	**RNNs**
Using	Typically used to extract spatial features from data in traffic prediction.	Designed to handle sequential and time-series data. Traffic is a dynamic and evolving system with temporal dependencies.
Traffic flow prediction	Process traffic flow data from sensors placed across different regions (such as traffic cameras and vehicle detectors), identifying patterns and trends in the spatial distribution of traffic.	By learning patterns from past traffic data (speed, volume, etc.), RNNs and LSTMs can forecast future traffic conditions.
Predicting congestion	In V2X systems with cameras or visual data from vehicles, CNNs can analyze images or video streams to detect traffic congestion, accidents, or roadblocks.	These models can predict future congestion or incidents based on the historical sequence of events in a given area.
Infrastructure	Process satellite or drone images to assess the conditions of roads or detect infrastructure events such as construction zones or accidents.	Used to predict the travel time for a given route, accounting for current and historical traffic conditions.

## 4 Proposed traffic predictors

Several studies used deep neural network (DNN) approaches to predict outcomes based on historical data (Abdellah et al., 2022b), including NARX (https://www.gpsworld.com/esa-backed-autonomous-driving-lab-coming-to-italy/), XGBoost (Yan et al., 2022), LSTM (Ban et al., 2022), and others. These studies predicted only procedural decisions early. They were also limited by (i) inadequate predictor selection for decision-making and (ii) the inability of conventional encoders to process the correlation of predictors in legal data.

This study proposed DL-based MSE, MAE, and SSE with Adam, SGDM, and RMSprop, tested with RNN and CNN models for V2X traffic prediction on V2X datasets. All simulations and programming have been conducted using MATLAB software. Initially, the V2X system was simulated to create the DL training dataset. Then, before the training phase, the collected V2X dataset was assessed, cleaned, and fed to the DL model for prediction. The DL model used 70% for the training set and 30% for the testing set. Normalize input data by maximum and lowest values to [0, 1]. The network receives the training dataset and a loss function, according to delta rules, adjusts the weights to reduce the error between observed and predicted outputs. After fitting the training models, the gradient of the loss function was determined, and the network weights and biases were adjusted. This procedure was continued until the output error was as small as possible. The test network requires test groups to evaluate the estimated model in the following phase.

**Figure 2:**
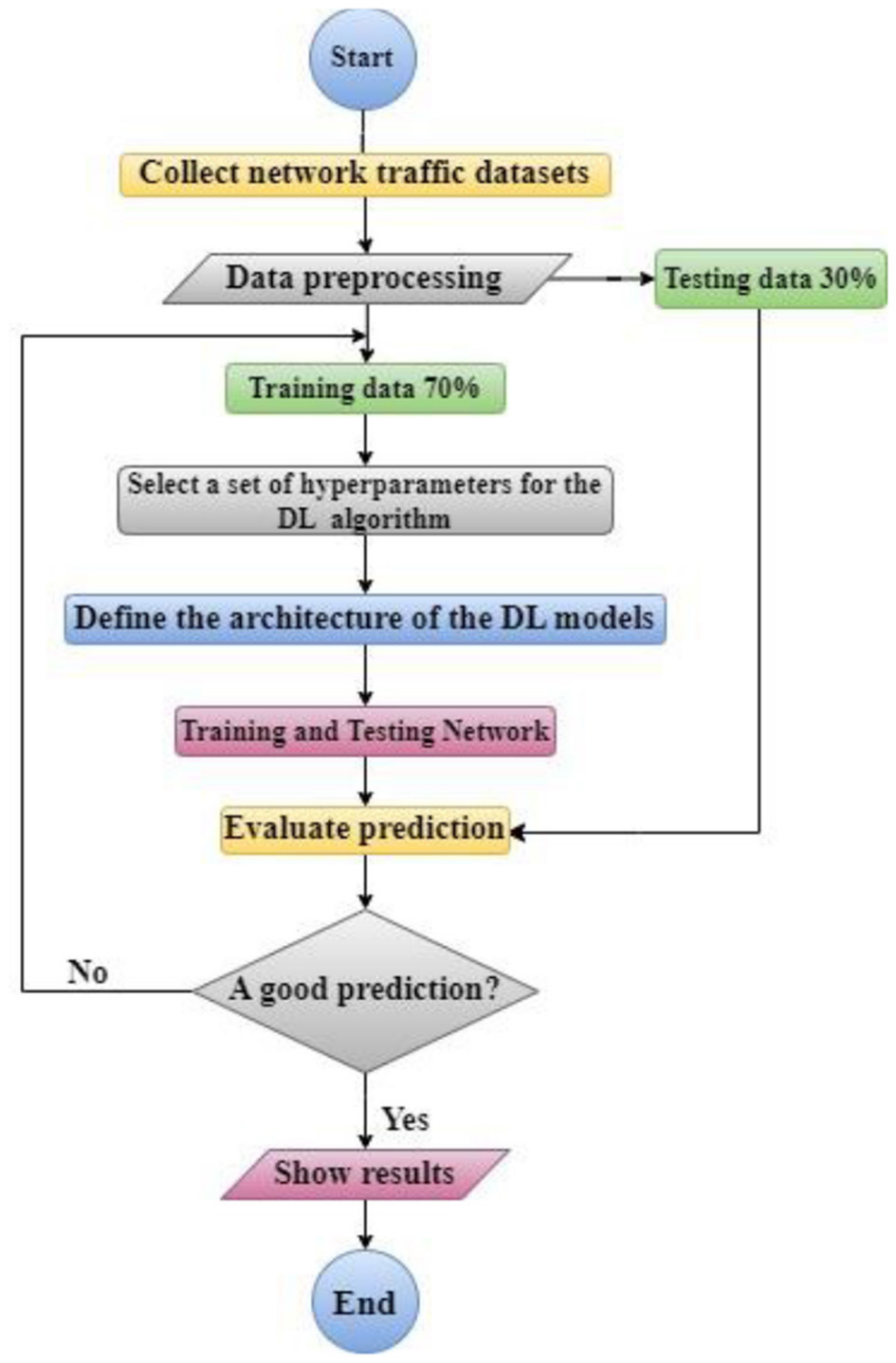
Flowchart for the proposed model.

For the first RNN model, when using traditional optimization techniques (Adam), a comparative study between the proposed BiLSTM and GRU models and the traditional LSTM model (Abdellah et al., 2022a) performance using RMSE and total FLOPS shows that the proposed models provide outstanding results for the desired application. Figure 2 shows the flowchart for the proposed work.

### 4.1 DL dataset collection and preprocessing

**Figure 3:**
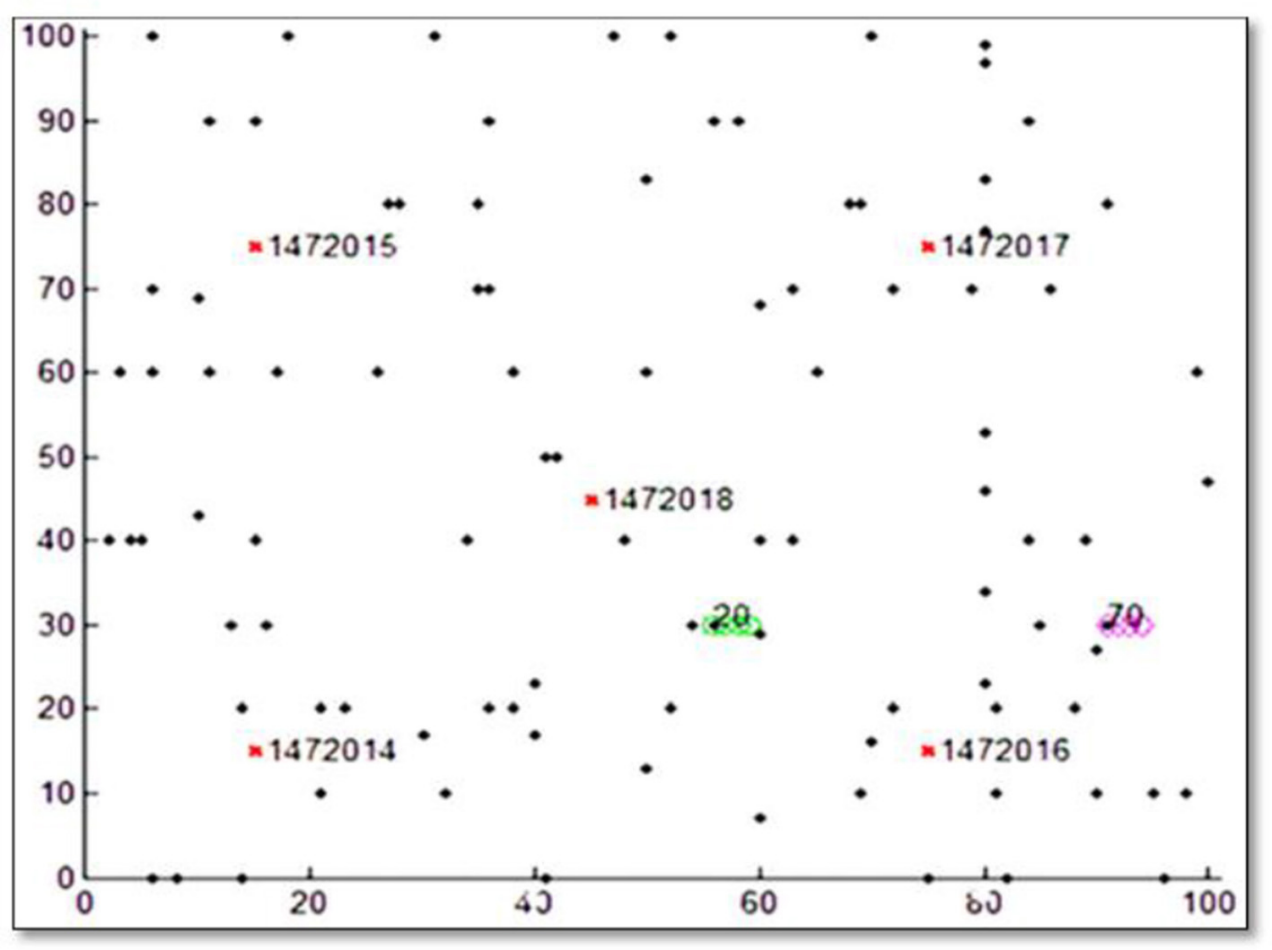
V2X diagram (Abdellah et al., 2022a).

This work collected a DL model training dataset from the V2X system (Abdellah et al., 2022a). All simulations and programming have been conducted in MATLAB software. Figure 3 shows the simulated V2X system. The simulation model is a V2X system for a smart city. Assume the size of the city on the x-y axis is 100 × 100. The mobility model helps city border nodes follow a fixed path in any direction. The dots in the figure represent Road Side Unit (RSU) nodes and positions, identified by network structure and configuration numbers. The model begins and ends at nodes 20 and 70. The simulation module visualizes network architecture and sets start and finish times. Randomly moving nodes can connect to distant nodes due to the RSUs' positions on the simulated map. RSUs can communicate with moving vehicles to send traffic information and safety alerts.

The training dataset size is 130 samples (130 × 1). QoS is known to be a critical and indispensable issue in networks. Therefore, one of the essential QoS parameters is throughput, which we collected and used as the dataset for training the ML model with the flow rules that generate it. In this work, we have made time-series predictions for V2X traffic, and the corresponding throughput is used as input to the ML training model. Data must be organized in a specific way for a time-series forecasting scenario, taking into account the data quality and the model used for data preparation. Data cleaning was used to eliminate tainted data and manage missing values. The data were then normalized to ensure all input parameters were within the range [0, 1]. The ML training leverages the network data that has been gathered. The network is trained using parameters such as the network architecture, training technique, and a minimum allowed error between the predicted and produced outputs. The trained ML's generalizability is checked: Unknown data are used to test the trained network, and the results are then compared to the actual results. The output data are post-processed: After the trained network has produced an acceptable result, the predicted value is post-processed to determine the output's normal value.

## 5 Simulation results

In this study, we want to improve the learning process to get a robust model. Therefore, we use various DLNN designs using different loss functions in the regression layer that will be trained in various cases, depending on the number of sent packets per second (4, 6, 8, 12, and 14 in a V2X environment). This will be done by replacing traditional loss functions, such as MSE, with more robust loss functions that will help in obtaining robust learning networks with better performance, especially in the presence of different forms of noise. The prediction accuracy regarding RMSE and total FLOPS will be evaluated. The proposed methodology is often compared to existing models using these evaluation parameters (comparing traffic flow forecast models). The lowest error value will be used to assess model performance in diverse contexts. The best predictor will be used for improving the QoS demands, monitoring resource management, enhancing security, and other operational issues. The DLNN parameters in each network are: epochs = 1,000, learning rate = 0.1, hidden layers = 50, and batch size = 16.

First, we use the traditional (default) loss function, mean squared error (MSE). The degree of inaccuracy in statistical models is measured by MSE. The average squared difference between the observed and anticipated values is evaluated. The MSE is equal to 0 in a model that has no errors. The value of the model inaccuracy increases with the error. The mean squared deviation (MSD), which is another name for the mean squared error, is calculated similarly to the variance. To determine the MSE, take the observed value, subtract the predicted value, and square that difference. Repeat that for all observations. Then, sum all of those squared values and divide by the number of observations. The numerator is the sum of the squared errors (SSE), which linear regression minimizes. MSE simply divides the SSE by the sample size. The formula for MSE is the following:


$$\begin{array}{l} MSE=\frac{{\sum \left(y_{i}-\hat{y_{i}}\right)}^{2}}{n} \end{array}$$ (1)

where

- *y*_*i*_ is the *i*th observed value,
- *y*_*i*_ is the corresponding predicted value, and
- *n* = the number of observations.

The MSE function has only one global minimum, with no local minimum, and it penalizes the model for making larger errors by squaring them. In contrast, the outliers are not handled properly, as the outlier error will be quite large, and it is penalized by squaring it.

Second, we built a custom regression layer that employs other loss functions such as “mean absolute error (MAE), sum of squared errors (SSE), Cauchy, Huber, etc.” to get the best network performance. Our second model was built using mean absolute error as a loss function. MAE is a popular metric with root mean squared error (RMSE); the error value units match the predicted target value units. Unlike RMSE, the changes in MAE are linear and therefore intuitive. MSE and RMSE penalize larger errors more, inflating or increasing the mean error value due to the square of the error value. In MAE, different errors are not weighted more or less, but the scores increase linearly with the increase in errors. The MAE score is measured as the average of the absolute error values. The absolute function is a mathematical function that makes a number positive. Therefore, the difference between an expected value and a predicted value can be positive or negative and will necessarily be positive when calculating the MAE. The MAE value can be calculated as follows:


$$\begin{array}{l} MAE=\frac{1}{n}\overset{n}{\underset{i=1}{\sum }}|y_{i}-\hat{y_{i}}{|}^{2} \end{array}$$ (2)

The advantage of MAE is that the outliers are handled better than MSE, as it does not penalize the model by squaring the error value. In contrast, its drawbacks are that it is computationally expensive (uses the modulus operator function) and there may be a local minimum.

Third, the model was built using the sum of squared errors (SSE) or residual sum of squares (RSS), where residual means remaining or unexplained. SSE is the difference between the observed and predicted values; it measures performance according to the sum of squared errors. The SSE calculation uses the following formula:


$$\begin{array}{l} SSE=\overset{n}{\underset{i=1}{\sum }}\varepsilon_{i}^{2} \end{array}$$ (3)

where ε_*i*_ is the difference between the actual value of the dependent variable and the predicted value:


$$\begin{array}{l} \varepsilon_{i}=y_{i}-\hat{y_{i}} \end{array}$$ (4)

Regression analysis aims to minimize the SSE—the smaller the error, the better the regression's estimation power.

### 5.1 Study of the hyperparameters for RNN

In this section, we study the effects of the hyperparameters, which include loss functions (MSE, MAE, and SSE) and optimizers (Adam, SGDM, and RMSprop) on different RNN models (LSTM, BiLSTM, and GRU) for V2X traffic prediction. The performance is evaluated in terms of accuracy using RMSE, as shown in Tables 2–4, and with the system complexity (efficiency) measured in Floating-Point Operations Per Second (FLOPS) (MegaFLOPS) as Tables 5–7 show.

**Table 2:** Summarized comparison of V2X traffic prediction accuracy (RMSE) for different RNN models for the MSE loss function with different optimizers.

**Packets/s**	**Adam**			**SGDM**			**RMSprop**		
	**LSTM** ^a^	**BiLSTM**	**GRU**	**LSTM**	**BiLSTM**	**GRU**	**LSTM**	**BiLSTM**	**GRU**
4	**0.5427**	**0.5174**	**0.4735**	**5.744**	**2.2119**	4.9530	2.7554	2.5584	**2.3222**
6	0.6321	0.6207	0.61636	5.8993	2.5543	4.9715	**2.0838**	2.7157	2.9293
8	0.8175	0.79799	0.70262	6.1655	3.2386	**3.7030**	2.4492	2.722	3.1809
10	1.1675	0.7135	0.7157	6.3592	2.9470	3.9094	2.3884	**2.0512**	3.4092
12	1.2622	0.6995	0.5737	6.822	3.9835	4.9540	2.7458	2.9886	3.4105
14	1.4911	0.6152	0.6130	7.2019	2.4141	3.9094	2.4724	2.5618	3.4965

^a^Abdellah et al. (2022a). The bold values represent the best result for that prediction for both RNN and CNN.

**Table 3:** Summarized comparison of V2X traffic prediction accuracy (RMSE) for different RNN models for the MAE loss function with different optimizers.

**Packets/s**	**Adam**			**SGDM**			**RMSprop**		
	**LSTM**	**BiLSTM**	**GRU**	**LSTM**	**BiLSTM**	**GRU**	**LSTM**	**BiLSTM**	**GRU**
4	**1.4555**	1.3756	0.89812	6.6981	4.0114	**6.7148**	2.7159	**1.2073**	**0.4745**
6	2.3610	**1.0096**	0.96599	5.9979	3.9289	7.1734	2.6281	2.1051	0.6818
8	2.1744	1.1630	0.67348	7.4874	4.0024	7.2875	**1.2699**	2.1879	0.6655
10	1.8571	1.5047	0.98492	5.9952	3.8980	7.2437	3.6717	2.1064	0.6756
12	2.4177	1.9274	**0.64724**	**5.8346**	**3.7098**	7.3951	3.6563	2.2094	0.5760
14	1.8417	1.2558	0.99262	6.8274	3.8539	7.5790	2.4164	1.6351	0.4756

The bold values represent the best result for that prediction for both RNN and CNN.

**Table 4:** Summarized comparison of V2X traffic prediction accuracy (RMSE) for different RNN models for the SSE loss function with different optimizers.

**Packets/s**	**Adam**			**SGDM**			**RMSprop**		
	**LSTM**	**BiLSTM**	**GRU**	**LSTM**	**BiLSTM**	**GRU**	**LSTM**	**BiLSTM**	**GRU**
4	1.4639	0.6892	0.8643	6.9345	1.1700	0.79059	**1.4581**	**0.5260**	**0.4649**
6	**0.8963**	0.9145	0.8275	6.9570	1.3792	0.7545	1.7791	0.6739	0.4685
8	1.2880	0.8723	0.8753	6.1173	1.5208	0.9585	2.1375	0.5797	0.5125
10	2.9876	0.8080	0.7724	6.7613	**0.8562**	0.9670	1.5639	0.5485	0.6646
12	0.9337	0.7332	0.7946	**6.0364**	1.0821	0.9486	1.8033	0.7871	0.5823
14	1.0959	**0.6608**	**0.7592**	6.2735	1.1749	**0.68481**	2.0758	0.6428	0.4969

The bold values represent the best result for that prediction for both RNN and CNN.

**Table 5:** Summarized comparison of V2X traffic prediction efficiency (FLOPS) for different RNN models for the MSE loss function with different optimizers.

**Packets/s**	**Adam**			**SGDM**			**RMSprop**		
	**LSTM**	**BiLSTM**	**GRU**	**LSTM**	**BiLSTM**	**GRU**	**LSTM**	**BiLSTM**	**GRU**
4	0.314135	0.627338	0.235463	0.313880	0.627083	0.235208	0.313931	0.627134	0.235259
6	0.209559	0.418361	0.157111	0.209304	0.418106	0.156856	0.209355	0.418157	0.156907
8	0.157271	0.313873	0.117935	0.157016	0.313618	0.117680	0.157067	0.313669	0.117731
10	0.125898	0.251180	0.094429	0.125643	0.250925	0.094174	0.125694	0.250976	0.094225
12	0.104983	0.209384	0.078759	0.104728	0.209129	0.078504	0.104779	0.209180	0.078555
14	0.090044	0.179530	0.067566	0.089789	0.179275	0.067311	0.089840	0.179326	0.067362

**Table 6:** Summarized comparison of V2X traffic prediction efficiency (FLOPS) for different RNN models for the MAE loss function with different optimizers.

**Packets/s**	**Adam**			**SGDM**			**RMSprop**		
	**LSTM**	**BiLSTM**	**GRU**	**LSTM**	**BiLSTM**	**GRU**	**LSTM**	**BiLSTM**	**GRU**
4	0.313689	0.626892	0.235017	0.313434	0.626637	0.234762	0.313485	0.626688	0.234813
6	0.209261	0.418063	0.156813	0.209006	0.417808	0.156558	0.209057	0.417859	0.156609
8	0.157048	0.313649	0.117712	0.156793	0.313394	0.117457	0.156844	0.313445	0.117508
10	0.125720	0.251001	0.094251	0.125465	0.250746	0.093996	0.125516	0.250797	0.094047
12	0.104834	0.209235	0.078610	0.104579	0.208980	0.078355	0.104630	0.209031	0.078406
14	0.089916	0.179403	0.067438	0.089661	0.179148	0.067183	0.089712	0.179199	0.067234

**Table 7:** Summarized comparison of V2X traffic prediction efficiency (FLOPS) for different RNN models for the SSE loss function with different optimizers.

**Packets/s**	**Adam**			**SGDM**			**RMSprop**		
	**LSTM**	**BiLSTM**	**GRU**	**LSTM**	**BiLSTM**	**GRU**	**LSTM**	**BiLSTM**	**GRU**
4	0.313699	0.626902	0.235027	0.313444	0.626647	0.234772	0.313495	0.626698	0.234823
6	0.209269	0.418071	0.156821	0.209014	0.417816	0.156566	0.209068	0.417867	0.156617
8	0.157054	0.313655	0.117718	0.156799	0.313400	0.117463	0.156850	0.313451	0.117514
10	0.125724	0.251006	0.094256	0.125469	0.250751	0.094007	0.125520	0.250802	0.094052
12	0.104838	0.209239	0.078614	0.104583	0.208984	0.078359	0.104634	0.209035	0.078410
14	0.089920	0.179406	0.067442	0.089665	0.179151	0.067187	0.089716	0.179202	0.067238

### 5.2 Study of the hyperparameters for CNN

In this section, we study the performance of CNN-based models for V2X traffic prediction with hyper parameters include loss functions (MSE, MAE, and SSE) and optimizers (Adam, SGDM, and RMSprop) on three activation functions [Rectified Linear Unit (ReLU), Tanh, and Sigmoid], Tanh, and Sigmoid. The performance is also measured using RMSE, which is shown in Tables 8–10, and total Floating-point Operations Per Second (FLOPS) (MegaFLOPS) as shown in Tables 11–13.

**Table 8:** Summarized comparison of V2X traffic prediction accuracy (RMSE) for CNNs with different activation functions for the MSE loss function with different optimizers.

**Packets/s**	**Adam**			**SGDM**			**RMSprop**		
	**ReLU**	**Tanh**	**Sigmoid**	**ReLU**	**Tanh**	**Sigmoid**	**ReLU**	**Tanh**	**Sigmoid**
4	**0.8871**	**0.78091**	0.94315	0.8511	**0.8484**	0.84087	0.9206	0.93295	**0.8883**
6	0.97617	0.87869	0.96466	0.89338	0.87815	0.83991	1.1531	0.87206	0.9709
8	1.1593	0.97886	0.94258	0.8655	0.87469	0.84007	**0.77211**	**0.78767**	0.9618
10	1.2904	0.87663	0.88514	0.8582	0.85902	**0.81922**	0.9121	0.89237	0.91306
12	1.1758	0.88064	0.8445	0.8781	0.88041	0.85139	0.97624	0.85949	0.94411
14	0.89422	0.88899	**0.84429**	**0.82728**	0.87465	0.86193	1.1237	0.85016	0.97612

The bold values represent the best result for that prediction for both RNN and CNN.

**Table 9:** Summarized comparison of V2X traffic prediction accuracy (RMSE) for CNNs with different activation functions for the MAE loss function with different optimizers.

**Packets/s**	**Adam**			**SGDM**			**RMSprop**		
	**ReLU**	**Tanh**	**Sigmoid**	**ReLU**	**Tanh**	**Sigmoid**	**ReLU**	**Tanh**	**Sigmoid**
4	0.87873	0.99886	0.84639	**1.0296**	0.98354	**1.7139**	0.94168	**0.80624**	0.88283
6	0.88119	0.93218	0.89103	1.2233	**0.97306**	16.8028	**0.91499**	0.87773	0.87897
8	0.90956	**0.84762**	0.86455	1.7913	1.0759	16.2821	1.0305	0.92576	0.87563
10	**0.87685**	0.86501	**0.84387**	1.6864	1.0635	24.7089	0.88992	0.8688	**0.85666**
12	0.90803	0.89081	0.88538	1.6287	1.0165	21.5408	0.87662	0.90726	0.86472
14	0.99185	0.91712	0.89195	1.7829	0.99035	4.0976	1.0323	0.89448	0.87001

The bold values represent the best result for that prediction for both RNN and CNN.

**Table 10:** Summarized comparison of V2X traffic prediction accuracy (RMSE) for CNNs with different activation functions for the SSE loss function with different optimizers.

**Packets/s**	**Adam**			**SGDM**			**RMSprop**		
	**ReLU**	**Tanh**	**Sigmoid**	**ReLU**	**Tanh**	**Sigmoid**	**ReLU**	**Tanh**	**Sigmoid**
4	**0.85166**	0.84635	0.97641	0.89092	**0.84165**	**0.82191**	0.94596	0.86342	0.92848
6	0.93447	0.87831	0.89499	0.88367	0.85108	0.83753	0.89885	0.84616	0.89532
8	0.87481	**0.77531**	0.85286	**0.78036**	0.88565	0.83854	1.383	**0.80995**	0.98149
10	0.88106	0.87322	**0.84953**	0.80463	0.86407	0.8458	0.85421	0.81993	**0.85517**
12	0.88894	0.8795	0.9503	0.84012	0.8668	0.85215	**0.8111**	0.8832	0.92741
14	0.8874	0.78672	0.87751	0.85073	0.8553	0.84721	0.94959	0.81997	0.96795

The bold values represent the best result for that prediction for both RNN and CNN.

**Table 11:** Summarized comparison of V2X traffic prediction efficiency (FLOPS) for CNNs with different activation functions for the MSE loss function with different optimizers.

**Packets/s**	**Adam**			**SGDM**			**RMSprop**		
	**ReLU**	**Tanh**	**Sigmoid**	**ReLU**	**Tanh**	**Sigmoid**	**ReLU**	**Tanh**	**Sigmoid**
4	0.011024	0.014936	0.012980	0.007268	0.011180	0.009224	0.009772	0.026680	0.011728
6	0.010196	0.013298	0.011747	0.006440	0.009542	0.007991	0.008944	0.026486	0.010495
8	0.009782	0.012478	0.011130	0.006026	0.008722	0.007374	0.008530	0.026388	0.009878
10	0.009534	0.011987	0.010760	0.005778	0.008231	0.007004	0.008282	0.026330	0.009508
12	0.009368	0.011659	0.010513	0.005612	0.007903	0.006757	0.008116	0.026291	0.009261
14	0.009250	0.011425	0.010337	0.005494	0.007669	0.006581	0.007998	0.026263	0.009085

**Table 12:** Summarized comparison of V2X traffic prediction efficiency (FLOPS) for CNNs with different activation functions for the MAE loss function with different optimizers.

**Packets/s**	**Adam**			**SGDM**			**RMSprop**		
	**ReLU**	**Tanh**	**Sigmoid**	**ReLU**	**Tanh**	**Sigmoid**	**ReLU**	**Tanh**	**Sigmoid**
4	0.010578	0.014490	0.012534	0.006822	0.010734	0.008778	0.009326	0.013238	0.011282
6	0.009899	0.013000	0.011449	0.006143	0.009244	0.007693	0.008647	0.011748	0.010197
8	0.009559	0.012255	0.010907	0.005803	0.008499	0.007151	0.008307	0.011003	0.009655
10	0.009355	0.011808	0.010582	0.005599	0.008052	0.006826	0.008103	0.010556	0.009330
12	0.009219	0.011510	0.010365	0.005463	0.007754	0.006609	0.007967	0.010258	0.009113
14	0.009122	0.011297	0.010210	0.005366	0.007541	0.006454	0.007870	0.010045	0.008958

**Table 13:** Summarized comparison of V2X traffic prediction efficiency (FLOPS) for CNNs with different activation functions for the SSE loss function with different optimizers.

**Packets/s**	**Adam**			**SGDM**			**RMSprop**		
	**ReLU**	**Tanh**	**Sigmoid**	**ReLU**	**Tanh**	**Sigmoid**	**ReLU**	**Tanh**	**Sigmoid**
4	0.010588	0.014500	0.012544	0.006832	0.010744	0.008788	0.009336	0.013248	0.011292
6	0.009906	0.013007	0.011457	0.006150	0.009251	0.007701	0.008654	0.011755	0.010205
8	0.009565	0.012261	0.010913	0.005809	0.008505	0.007157	0.008313	0.011008	0.009661
10	0.009360	0.011813	0.010586	0.005604	0.008057	0.006830	0.008108	0.010561	0.009334
12	0.009223	0.011514	0.010369	0.005467	0.007758	0.006613	0.007971	0.010262	0.009117
14	0.009126	0.011301	0.010213	0.005370	0.007545	0.006457	0.007874	0.010049	0.008961

According to the results listed in Tables 2–7 for our RNN models, for the Adam optimizer with MSE loss function, the results indicate that for lower packet rates (4 packets/s), GRU performs best with the lowest RMSE of 0.4735 and a reasonable computational cost of 235,463 FLOPS. As the packet rate increases, GRU consistently shows lower RMSE values compared to LSTM and BiLSTM, making it the most efficient model in terms of both prediction accuracy and computational cost (complexity). With the MAE loss function, the performance is similar to the GRU model, exhibiting the best balance between accuracy and efficiency. With SSE loss function results also show that GRU generally performs better, followed by the BiLSTM model, but the BiLSTM has more complexity than the GRU model. For the SGDM optimizer, the observed trends resemble those associated with the Adam optimizer; however, they are characterized by comparatively elevated RMSE values and increased computational costs. For the MSE loss function, the BiLSTM model performs the best at 4 packets/s, with an RMSE of 2.2119, although it requires significantly more computational resources (627,083 FLOPS) than the GRU. With MAE, the worst performance is observed. With SSE, the GRU performs best compared to LSTM and BiLSTM. For the RMSprop optimizer, results show that compared with Adam and SGDM, RMSprop generally shows lower RMSE values across all models. In MSE, the results are not as good compared with other loss functions; in contrast, RMSprop with SSE yields better RMSE with BiLSTM and GRU. In summary, the overall results from RNN analysis indicate that the GRU model outperforms both LSTM and BiLSTM models in terms of prediction accuracy (RMSE) and efficiency (FLOPS). This superiority is particularly clear with MSE as the loss function, followed by MAE. Also, it is essential to recognize that the selection of the optimizer plays a crucial role, with Adam generally yielding the best performance across the different loss functions.

The results for the CNN models (Tables 8–13) show the following:

For the Adam optimizer with the MSE loss function, the Tanh activation function generally provides the best trade-off between prediction accuracy and system complexity (computational cost) across all packet rates, with the lowest RMSE value of 0.78091 at 4 packets/s and 14,936 FLOPS. All three activation functions perform well with the MAE loss function, with only slight variations between them. With the SSE loss function, the performance trend is similar, with Tanh achieving the lowest RMSE values and a good balance between accuracy and efficiency.

For SGDM, performance with MSE and SSE is similar to the Adam optimizer, but the MAE loss function yields poor results. However, SGDM requires fewer computational resources than Adam. For MSE, at a higher data rate, the Sigmoid and then ReLU activation functions achieve the best accuracy compared with the Tanh activation function. The MAE loss function indicates that the Tanh activation function typically yields less accuracy than the ReLU and Sigmoid activation functions, but it requires more computational resources. With the SSE loss function, ReLU achieves a good balance between accuracy and efficiency, especially with 8 packets/s, followed by Sigmoid.

The performance of CNN models using the RMSprop optimizer is close to that of Adam and SGDM. For the three loss functions, the Tanh activation function yields the best performance in terms of accuracy. In contrast, the computational cost is relatively high, which highlights the trade-off between accuracy and computational efficiency with RMSprop.

In general, for CNN results, we can conclude that ReLU generally outperforms other activation functions (Tanh and Sigmoid) across different loss functions and optimizers in terms of both RMSE and computational efficiency. Although Tanh yields the best accuracy, it comes with high computational resources. The Sigmoid activation function offers a middle ground for accuracy and efficiency. The Adam optimizer typically results in the best overall performance, especially with Tanh in terms of accuracy, but with ReLU providing the most efficient model in terms of both accuracy and computational cost.

**Figure 4:**
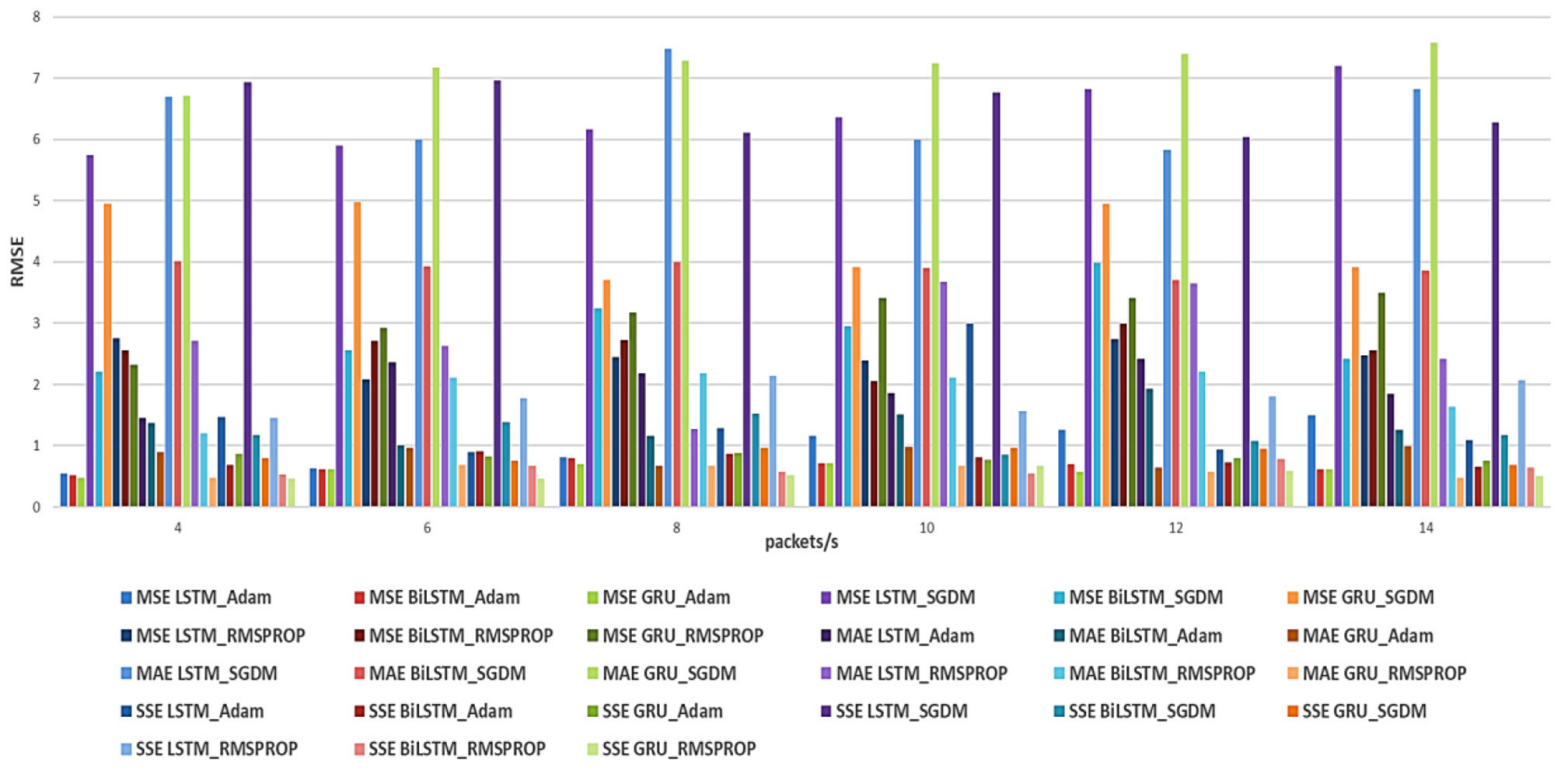
Summarized RMSE for the three loss functions (MSE, MAE, and SSE) with different optimizers for RNN models.

**Figure 5:**
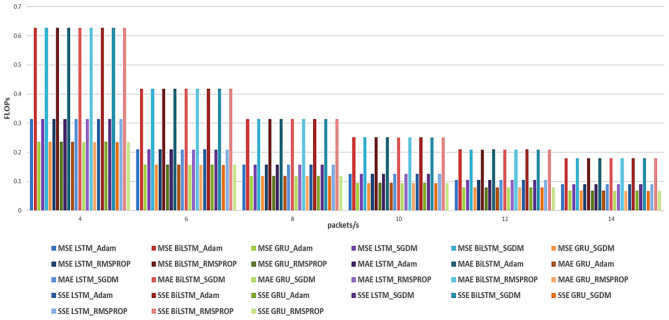
Summarized total FLOPS for the three loss functions (MSE, MAE, and SSE) with different optimizers for RNN models.

**Figure 6:**
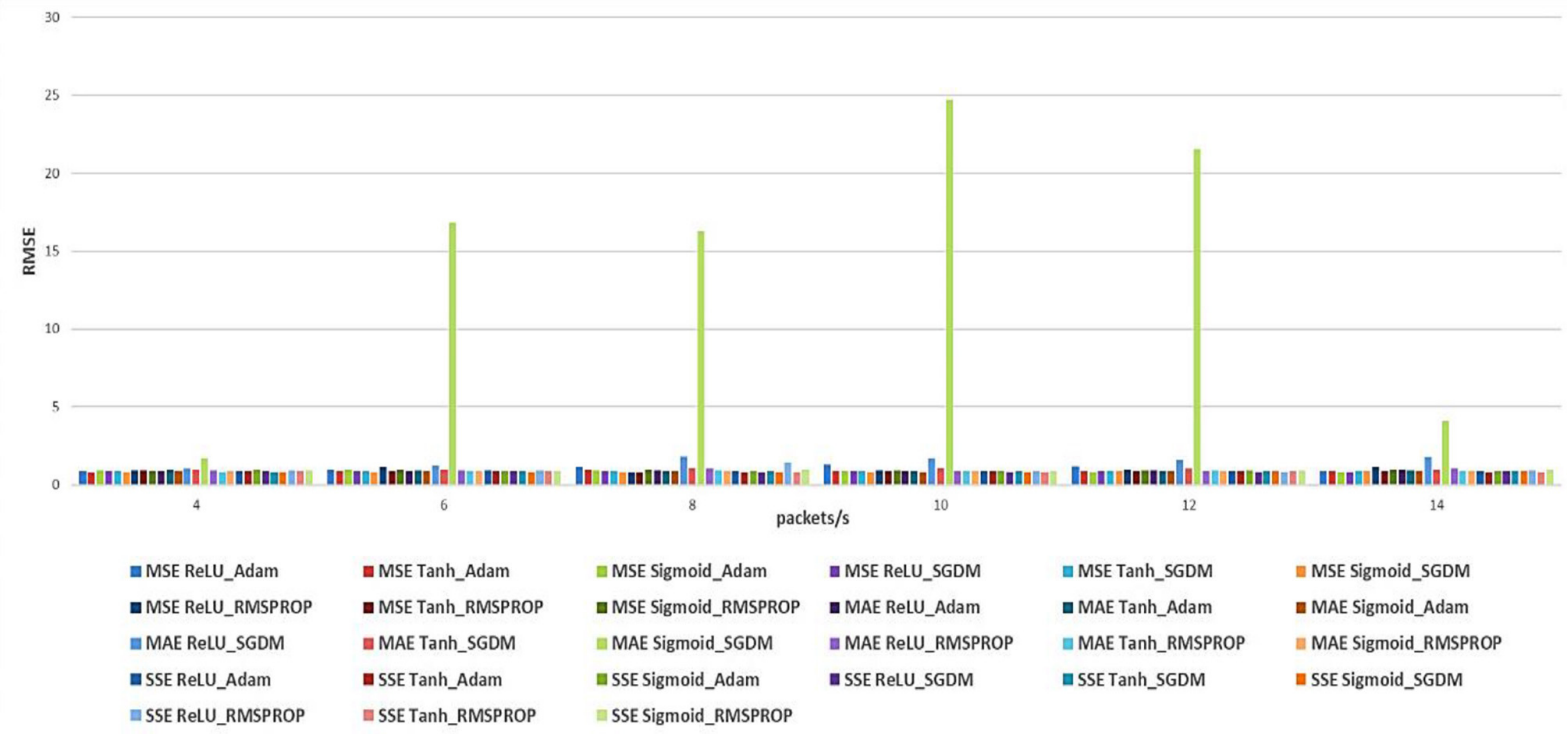
Summarized RMSE for the three loss functions (MSE, MAE, and SSE) with different optimizers and different activation functions for CNN models.

**Figure 7:**
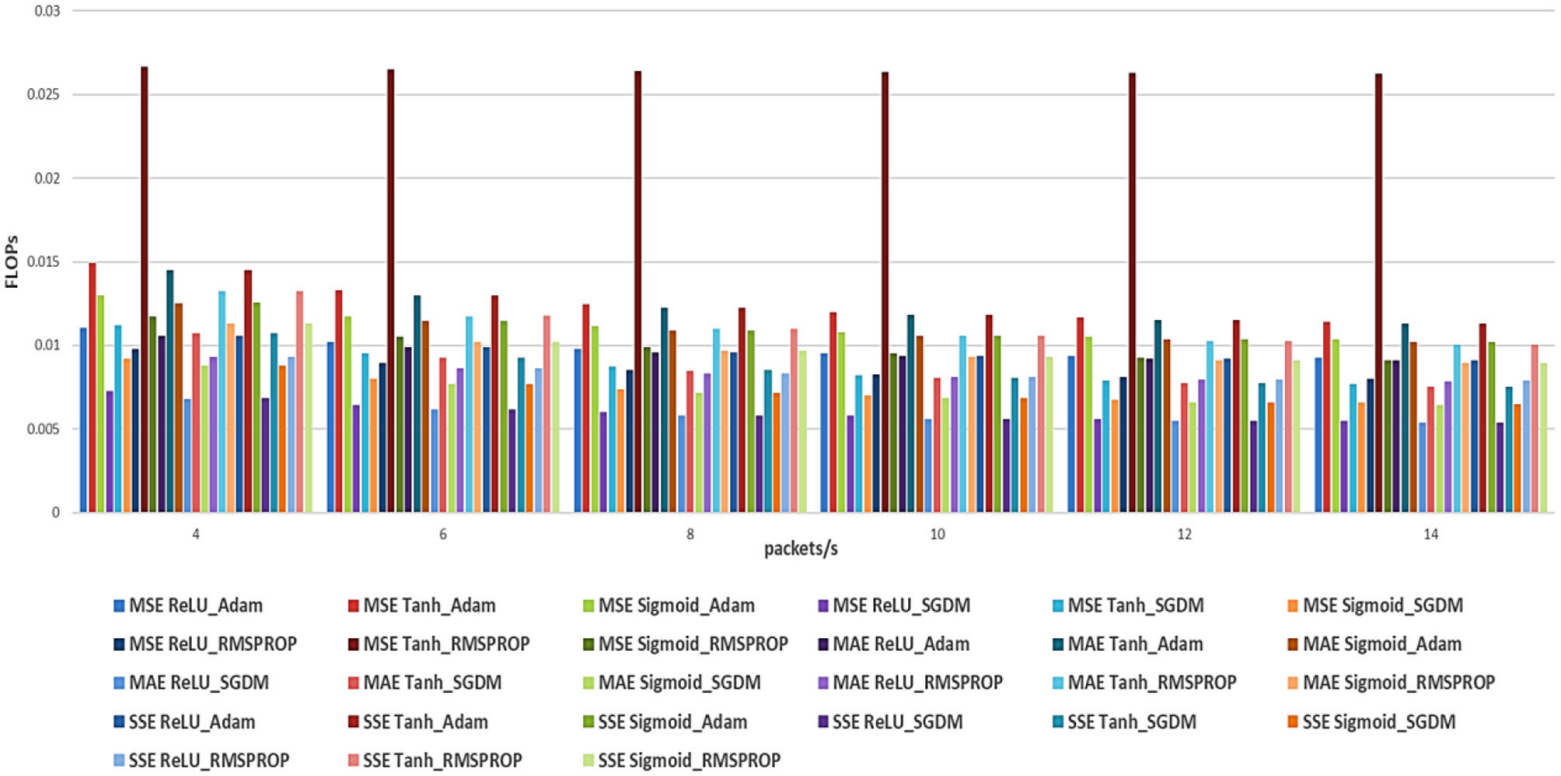
Summarized total FLOPS for the three loss functions (MSE, MAE, and SSE) with different optimizers and different activation functions for CNN models.

Figures 4, 5 show the summary of RMSE and FLOPS, respectively, according to the packet rate for the Recurrent Neural Network models, and Figures 6, 7 show the summary for Convolutional Neural Network models as well.

The analysis of RNN and CNN models in the context of V2X traffic prediction reveals that each model type has its advantages depending on the use case. The RNN models, particularly the Gated Recurrent Unit (GRU), outperform CNNs in terms of prediction accuracy (lower RMSE values), but in contrast, they need more computational resources (higher FLOPS). In contrast, CNN models generally demand less computational resources, especially when employing Rectified Linear Unit (ReLU) activation functions and the Adam optimizer, which makes them a more efficient choice for applications where computational cost is a critical factor. Although CNNs may not consistently reach the low RMSE values that RNNs can achieve, they offer a favorable balance between accuracy and computational efficiency. Ultimately, the decision to choose between RNN and CNN models depends on the specific requirements of the traffic prediction task, such as the required accuracy, computational resources, and packet rate.

Table 14 shows the comparison of the proposed RNN and CNN models with related work.

**Table 14:** Comparison of model performance and related works.

**Model**	**Packet rate**	**RMSE**	**Optimizer**	**Loss function**	**Computational efficiency**	**Comparison with related work**	**Key insights**
BiLSTM and GRU	4 packets/s	**0.5174 and 0.4735** Vs. 0.5427	Adam	MSE	Low FLOPS	Achieves the lowest RMSE, outperforming existing LSTM-based approaches in Abdellah et al. (2022a).	Achieves better performance than LSTM in terms of efficiency and accuracy
GRU	4 packets/s	**0.4735** vs. 0.604	Adam	MSE	Low FLOPS	Comparable to Kim et al. (2021); better RMSE and reduced computational cost	Outperforms LSTM, BiLSTM in accuracy and efficiency
LSTM	4 packets/s	**5.744** vs. 5.78	SGDM	MSE	High FLOPS	Similar to Mahajan et al. (2024); struggles with noisy, variable traffic data	LSTM requires optimization tuning for improved performance
CNN (ReLU)	4 packets/s	**0.8871** vs. 0.962	Adam	MSE	Moderate FLOPS	Comparable to Cao et al. (2021); outperforms the hybrid CNN-LSTM model	Efficient, simpler approach compared to hybrid CNN-LSTM
CNN (ReLU)	4 packets/s	**0.8871** vs. 0.98	Adam	MSE	Lower FLOPS	Better than Ma et al. (2017); requires fewer resources	Shows potential for real-time prediction with fewer parameters
GRU	–	–	–	SSE	–	Contrasts with the typical preference for MSE in traffic prediction tasks	SSE is more effective for minimizing large prediction errors
RNN and CNN	–	–	Adam	–	–	Adam optimizer outperforms SGDM and RMSprop in the literature	Adaptive optimizers yield superior performance in both accuracy and efficiency

The bold values represent the best result for that prediction for both RNN and CNN.

## 6 Robustness to noise in real-world V2X scenarios

The reviewer's comment rightly highlights a critical aspect of deploying deep learning models in practical settings: robustness to noise. Real-world V2X communication channels are susceptible to various impairments, including signal fading, multi-path propagation, interference from other devices, and sensor inaccuracies (Al-Qatf et al., 2018). These factors introduce noise and uncertainties into the data stream, which can significantly degrade the performance of prediction models that were trained on clean or idealized datasets.

### 6.1 Impact of noise on prediction models

The presence of noise in the input data can have several detrimental effects on traffic prediction models:

- The most direct impact is an increase in prediction error. Noisy inputs obscure the underlying temporal patterns and relationships that models like RNNs and CNNs are designed to learn. This can lead to a substantial rise in RMSE, rendering the predictions less reliable for critical tasks like collision avoidance or traffic optimization.
- Deep neural networks have a high capacity to learn complex patterns, which can lead them to inadvertently memorize the noise in the training data as if it were a genuine feature. A model overfitted in this way will perform poorly when deployed, as the real-world noise characteristics will differ from those in the training set.
- A model that has not been exposed to noisy conditions during training may fail to generalize to different operational environments (e.g., urban canyons, rural areas, and adverse weather) where the channel quality and noise profiles vary.

### 6.2 Evaluating model robustness: a proposed framework

To ensure the practical viability of our proposed DLNN predictors, it is imperative to evaluate their robustness. A standard approach is to test the trained models on a dataset corrupted with synthetic noise that mimics real-world channel impairments, such as Additive White Gaussian Noise (AWGN).

The robustness can be quantified by observing the rate of performance degradation as the noise level increases. Key metrics for this analysis include:

- Root mean squared error (RMSE)**:** Monitoring the increase in RMSE as a function of the Signal-to-Noise Ratio (SNR).
- Normalized mean squared error (NMSE): This provides a normalized measure of the deviation, making it easier to compare performance across different models and noise levels.

A robust model will exhibit a slower increase in RMSE/NMSE and maintain acceptable prediction accuracy even under moderate-to-high levels of noise.

### 6.3 Inherent robustness of the proposed models

Our proposed architecture, particularly the RNN variants (LSTM, BiLSTM, GRU), possesses inherent characteristics that can contribute to noise robustness:

- The GRU and LSTM units are specifically designed to handle long-term dependencies and can learn to “forget” or “ignore” short-term, uncorrelated fluctuations (i.e., noise) while focusing on the more persistent, underlying trends in the traffic data. The gating mechanisms allow these models to regulate the flow of information, potentially filtering out noisy inputs.
- By processing sequences of data points, RNNs inherently perform a form of temporal smoothing. A single noisy data point within a sequence has a diminished impact on the overall prediction, as the output is based on the context of the entire sequence.

While CNNs are powerful for feature extraction, their robustness to temporal noise can be lower than that of RNNs unless they are specifically regularized or trained with noisy data, as they primarily capture spatial or local temporal patterns.

### 6.4 Mitigation strategies and future work

To further enhance robustness, several strategies can be employed, which also form a basis for our immediate future work:

- Introducing controlled levels of AWGN during the training phase itself. This acts as a powerful regularization technique, forcing the model to learn features that are invariant to small perturbations and preventing overfitting to clean data (Sepasgozar and Pierre, 2022).
- As explored in this paper, certain loss functions like MAE are inherently more robust to outliers than MSE. Deploying such loss functions in noisy environments could lead to more stable models.
- Implementing pre-processing filters (e.g., Kalman filters and moving average filters) on the input V2X data stream before it is fed into the prediction model.

While the core results presented in Section 5 demonstrate the superior performance of our GRU and CNN models under clean data conditions, we acknowledge that robustness to noise is a non-negotiable requirement for real-world V2X deployment. The architectural advantages of RNNs, particularly GRUs, suggest a natural resilience to noisy inputs. A comprehensive evaluation of this robustness, following the framework outlined above, will be a central focus of our subsequent research to transition these models from a theoretical benchmark to a practical solution for intelligent transportation systems.

## 7 Conclusion

This paper presents a comprehensive investigation into the DLNN models, specifically RNNs (LSTM, BiLSTM, and GRU) and CNNs, for accurate and efficient V2X traffic prediction. We explored the impact of various hyperparameters, including loss functions (MSE, MAE, and SSE) and optimizers (Adam, SGDM, and RMSprop), on the performance of these models. The proposed predictor uses past traffic data to predict future traffic patterns to improve forecasting and decision-making in V2X networks. The prediction problems are studied in different cases depending on the number of packets sent per second. The prediction accuracy is measured in terms of RMSE and the number of FLOPS. A critical finding of this study is the evaluation of the models' robustness under simulated real-world conditions. By testing the models against data corrupted with Additive White Gaussian Noise (AWGN), which mimics sensor imprecision and channel impairments inherent to V2X communication, the following was established: the noise generally increased the prediction error (RMSE) across all models, confirming its detrimental effect on input data quality. However, the recurrent architectures, particularly the GRU and BiLSTM, demonstrated superior resilience, exhibiting a slower degradation rate in accuracy compared to the CNN models as the noise level increased. This inherent ability to filter noise, due to the sequential nature and gating mechanisms of RNNs, validates the use of these DLNNs as a practically viable solution for proactive traffic management systems that rely on potentially noisy V2X data streams.

In conclusion, the GRU model is the recommended choice for V2X traffic prediction, offering the best trade-off between high accuracy, low computational complexity, and essential robustness against real-world data noise. For the optimizer's impact, the Adam optimizer consistently outperformed SGDM and RMSprop in terms of both accuracy and efficiency. For the loss function effect, while MSE is a common choice, SSE can be advantageous in specific scenarios where minimizing large errors is crucial.

## Data Availability

The original contributions presented in the study are included in the article/supplementary material, further inquiries can be directed to the corresponding author.
