# Correction: Deep learning neural networks-based traffic predictors for V2X communication networks

**DOI:** 10.3389/frai.2025.1768205

**Published:** 2026-01-13

**Authors:** Marina Magdy Saady, Hatim Ghazi Zaini, Mohamed Hassan Essai Ali, Sahar A. El_Rahman, Osama A. Omer, Ali R. Abdellah, Shaima Elnazer

**Affiliations:** 1 Department of Electrical Engineering, Faculty of Engineering, Aswan University, Aswan, Egypt; 2 Computer Engineering Department, College of Computer and Information Technology, Taif University, Taif, Saudi Arabia; 3 Department of Electrical Engineering, Faculty of Engineering, Al-Azhar University, Qena, Egypt; 4 Department of Computer Systems Program-Electrical Engineering, Faculty of Engineering-Shoubra, Benha University, Cairo, Egypt; 5 Nile Higher Institute of Engineering and Technology, Mansoura, Egypt

**Keywords:** CNN, deep learning, optimizers, RNN, traffic prediction, V2X

The affiliation for Ali R. Abdellah was erroneously given as (5) Department of Electrical Engineering, Faculty of Engineering, Al-Azhar University, Qena/Aswan, Egypt.

This affiliation has been removed.

The correct affiliation for Ali R. Abdellah should: 3 Department of Electrical Engineering, Faculty of Engineering, Al-Azhar University, Qena, Egypt.

The affiliation listed for author Shaima Elnazer was erroneously given as (6). This should be: 5 Nile Higher Institute of Engineering and Technology, Mansoura, Egypt.

The original version of this article has been updated.

